# Supplementary material for: Cognitive ability and physical health: a Mendelian randomization study
Source: Sci Rep. 2017 Jun 1;7:2651. doi: 10.1038/s41598-017-02837-3 (PMC5453939; doi:10.1038/s41598-017-02837-3)
Supplement: Supplementary file 1 — Supplementary Material [file 41598_2017_2837_MOESM1_ESM.pdf]

Supplementary Materials for:

Cognitive ability and physical health: a Mendelian randomization study

Saskia P Hagenaars, Catharine R Gale, Ian J Deary and Sarah E Harris

Contents

Supplementary Figure 1: Educational attainment and verbal-numerical reasoning in UK

Biobank

Supplementary Table 1: Power calculations

Supplementary Table 2: Observational associations between educational attainment and health outcomes.

Supplementary Table 3a-f: Summary of the SNPs used for instrumental variants previously identified as associated with exposure at genome wide significance

Supplementary figure 1. Educational attainment categories and mean scores for verbal-numerical reasoning per group. Error bars respresent standard errors.

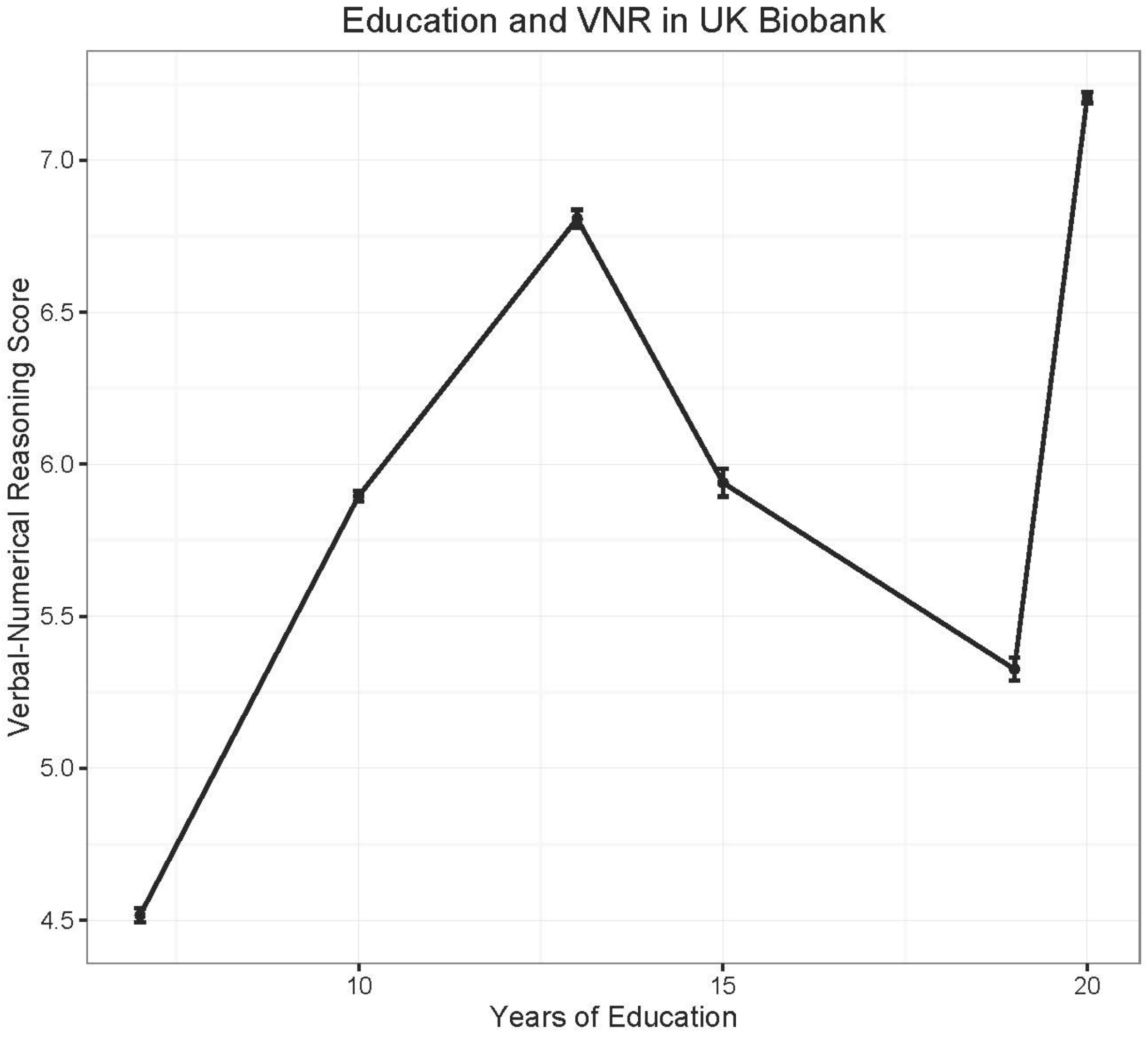

Supplementary Table 1. Power for bidirectional Mendelian randomization analysis (two-sided α = 0.05). Power calculations are based on the method developed by Brion, et al. [1].

Exposure/instrumental variable	Outcome	R-squared (of variance in exposure)	Actual N (outcome)	Proportion of cases (outcome)	Observational Effect size	N required for 80% power	Power at actual N
Educational attainment	BMI	0.015	111712	NA	-0.37	3299	1
Educational attainment	Height	0.015	111959	NA	0.31	4922	1
Educational attainment	Systolic blood pressure	0.015	106759	NA	-0.2	12559	1
Educational attainment	Coronary artery disease	0.015	110,072	0.048	OR 0.4	42,832	1
Educational attainment	Type 2 diabetes	0.015	111,779	0.034	OR 0.6	86,084	0.89
BMI	Verbal-numerical reasoning	0.027	36,035	NA	-0.05	115990	0.35
Height	Verbal-numerical reasoning	0.15	36,035	NA	0.18	1563	1
Systolic blood pressure	Verbal-numerical reasoning	0.009	36,035	NA	-0.05	347968	0.15
Coronary artery disease	Verbal-numerical reasoning	0.1	36,035	NA	-0.27	999	1
Type 2 diabetes	Verbal-numerical reasoning	0.057	36,035	NA	-0.06	36207	0.8

Supplementary Table 2. Observational associations between educational attainment and health outcomes.

	Educational attainment – health outcomes (7 vs 10 years)			Educational attainment – health outcomes (7 vs 13 years)		
	Beta	95% CI	p	Beta	95% CI	p
BMI	-0.1287	-0.146, -0.111	**7.25×10^{-44}**	-0.2350	-0.257, -0.213	**8.15×10^{-93}**
Height	0.1667	0.155, 0.178	**2.25×10^{-151}**	0.2590	0.243, 0.275	**1.94×10^{-235}**
Systolic blood pressure	-0.0775	-0.095, -0.060	**6.89×10^{-18}**	-0.1273	-0.149, -0.106	**1.71×10^{-30}**
Type 2 diabetes	OR: 0.815	0.742, 0.895	**1.74×10^{-05}**	OR: 0.800	0.706, 0.905	**4.27×10^{-04}**
Coronary artery disease	OR: 0.631	0.583, 0.683	**2.79×10^{-30}**	OR: 0.555	0.496, 0.620	**2.98×10^{-25}**

Supplementary Table 3a-f. Summary of the SNPs used for instrumental variants previously identified as associated with exposure at genome wide significance.

See attached Excel spreadsheet

References

1 Brion, M.-J. A., Shakhbazov, K. & Visscher, P. M. Calculating statistical power in Mendelian randomization studies. *International Journal of Epidemiology* **42**, 1497-1501, doi:10.1093/ije/dyt179 (2013).

Supplementary Table 3a: Summary of the BMI SNPs previously identified as associated with BMI at genome wide significance.

SNP	Chr	Position (bp)	A1	A2	Frequency	Beta	SE	P	Nearest Gene
rs1000940	17	5223976	G	A	0.225	0.0192	0.0034	1.28E-08	RABEP1
rs10132280	14	24998019	A	C	0.3333	-2.30E-02	0.0034	1.14E-11	STXBP6
rs1016287	2	59159129	T	C	0.325	2.29E-02	0.0034	2.25E-11	LINC01122
rs10182181	2	25003800	A	G	0.5	-0.0307	0.0031	8.78E-24	ADCY3
rs10733682	9	128500735	A	G	0.425	0.0174	0.0031	1.83E-08	LMX1B
rs10938397	4	44877284	A	G	0.5667	-0.0402	0.0031	3.21E-38	GNPDA2
rs10968576	9	28404339	G	A	0.2917	2.49E-02	0.0033	6.61E-14	LINGO2
rs11030104	11	27641093	A	G	0.8	4.14E-02	0.0038	5.56E-28	BDNF
rs11057405	12	121347850	A	G	0.0917	-3.07E-02	0.0055	2.02E-08	CLIP1
rs11126666	2	26782315	G	A	0.6917	-2.07E-02	0.0034	1.33E-09	KCNK3
rs11165643	1	96696685	C	T	0.425	-2.18E-02	0.0031	2.07E-12	PTBP2
rs11191560	10	104859028	T	C	0.9417	-0.0308	0.0053	8.45E-09	NT5C2
rs11583200	1	50332407	C	T	0.375	1.77E-02	0.0031	1.48E-08	ELAVL4
rs1167827	7	75001105	A	G	0.4583	-0.0202	0.0033	6.33E-10	HIP1
rs11688816	2	62906552	A	G	0.5417	-0.0172	0.0031	1.89E-08	EHBP1
rs11727676	4	145878514	C	T	0.075	-0.0358	0.0064	2.55E-08	HHIP
rs11847697	14	29584863	T	C	0.0417	4.92E-02	0.0084	3.99E-09	PRKD1
rs12286929	11	114527614	G	A	0.4333	0.0217	0.0031	1.31E-12	CADM1
rs12401738	1	78219349	A	G	0.425	2.11E-02	0.0033	1.15E-10	FUBP1
rs12429545	13	53000207	G	A	0.9	-0.0334	0.0047	1.09E-12	OLFM4
rs12446632	16	19842890	A	G	0.1333	-4.03E-02	0.0046	1.48E-18	GPRC5B
rs12566985	1	74774781	G	A	0.425	0.0242	0.0031	3.28E-15	FPGT-TNNI3K
rs12885454	14	28806589	C	A	0.6333	2.07E-02	0.0033	1.94E-10	PRKD1
rs12940622	17	76230166	A	G	0.4583	-1.82E-02	0.0031	2.49E-09	RPTOR
rs13021737	2	622348	A	G	0.125	-6.01E-02	0.004	1.11E-50	TMEM18
rs13078960	3	85890280	T	G	0.8167	-0.0297	0.0039	1.74E-14	CADM2
rs13107325	4	103407732	C	T	0.8833	-4.77E-02	0.0068	1.83E-12	SLC39A8
rs13191362	6	162953340	A	G	0.8	2.77E-02	0.0048	7.34E-09	PARK2
rs1516725	3	187306698	T	C	0.0917	-4.51E-02	0.0046	1.89E-22	ETV5
rs1528435	2	181259207	T	C	0.5833	0.0178	0.0031	1.20E-08	UBE2E3
rs16851483	3	142758126	G	T	0.9083	-4.83E-02	0.0077	3.55E-10	RASA2
rs16951275	15	65864222	C	T	0.225	-3.11E-02	0.0037	1.91E-17	MAP2K5
rs17024393	1	109956211	C	T	0.04167	0.0658	0.0088	7.03E-14	GNAT2
rs17094222	10	102385430	C	T	0.2083	0.0249	0.0038	5.94E-11	HIF1AN
rs17405819	8	76969139	C	T	0.3667	-0.0224	0.0033	2.07E-11	HNF4G
rs17724992	19	18315825	A	G	0.6917	0.0194	0.0035	3.42E-08	PGPEP1
rs1808579	18	19358886	T	C	0.475	-1.67E-02	0.0031	4.17E-08	C18orf8
rs1928295	9	119418304	C	T	0.425	-0.0188	0.0031	7.91E-10	TLR4
rs2033732	8	85242264	C	T	0.7583	0.0192	0.0035	4.89E-08	RALYL
rs205262	6	34671142	A	G	0.7333	-2.21E-02	0.0035	1.75E-10	C6orf106
rs2112347	5	75050998	G	T	0.375	-0.0261	0.0031	6.19E-17	POC5
rs2121279	2	142759755	T	C	0.1167	0.0245	0.0044	2.31E-08	LRP1B
rs2176598	11	43820854	T	C	0.2	0.0198	0.0036	2.97E-08	HSD17B12
rs2207139	6	50953449	G	A	0.1	4.47E-02	0.004	4.13E-29	TFAP2B
rs2245368	7	76446079	T	C	0.7583	-3.17E-02	0.0057	3.19E-08	PMS2L11
rs2287019	19	50894012	C	T	0.85	3.60E-02	0.0042	4.59E-18	QPCTL
rs2365389	3	61211502	C	T	0.6583	2.00E-02	0.0031	1.63E-10	FHIT
rs2650492	16	28240912	A	G	0.3083	2.07E-02	0.0035	1.92E-09	SBK1
rs2820292	1	200050910	A	C	0.4917	-0.0195	0.0031	1.83E-10	NAV1
rs29941	19	39001372	A	G	0.3333	-1.82E-02	0.0033	2.41E-08	KCTD15
rs3101336	1	72523773	T	C	0.3509	-3.34E-02	0.0031	2.66E-26	NEGR1
rs3736485	15	49535902	A	G	0.425	1.76E-02	0.0031	7.41E-09	DMXL2
rs3810291	19	52260843	A	G	0.625	0.0283	0.0036	4.81E-15	ZC3H4
rs3817334	11	47607569	C	T	0.55	-0.0262	0.0031	5.15E-17	MTCH2
rs3849570	3	81874802	A	C	0.3667	0.0188	0.0034	2.60E-08	GBE1
rs3888190	16	28796987	A	C	0.3583	3.09E-02	0.0031	3.14E-23	ATP2A1
rs4740619	9	15624326	T	C	0.5333	0.0179	0.0031	4.56E-09	C9orf93
rs543874	1	176156103	G	A	0.2667	4.82E-02	0.0039	2.62E-35	SEC16B
rs6477694	9	110972163	C	T	0.3583	0.0174	0.0031	2.67E-08	EPB41L4B
rs6567160	18	55980115	C	T	0.2833	5.56E-02	0.0036	3.93E-53	MC4R

rs657452	1	49362434	A	G	0.4167	0.0227	0.0031	5.48E-13	AGBL4
rs6804842	3	25081441	A	G	0.425	-1.85E-02	0.0031	2.48E-09	RARB
rs7138803	12	48533735	G	A	0.5583	-3.15E-02	0.0031	8.15E-24	BCDIN3D
rs7141420	14	78969207	T	C	0.6167	0.0235	0.0031	1.23E-14	NRXN3
rs7243357	18	55034299	G	T	0.1333	-2.17E-02	0.004	3.86E-08	GRP
rs758747	16	3567359	C	T	0.7333	-2.25E-02	0.0037	7.47E-10	NLRC3
rs7599312	2	213121476	G	A	0.7083	0.022	0.0034	1.17E-10	ERBB4
rs7899106	10	87400884	A	G	0.95	-3.95E-02	0.0071	2.96E-08	GRID1
rs7903146	10	114748339	T	C	0.25	-2.34E-02	0.0034	1.11E-11	TCF7L2
rs9400239	6	109084356	C	T	0.7	1.88E-02	0.0033	1.61E-08	FOXO3

Supplementary Table 3b: Summary of the height SNPs previously identified as associated with height at genome wide significance

SNP	Chr	Position (bp)	A1	A2	Frequency A1	Beta	SE	P	Nearest Gene
rs10883563	10	102674370	A	C	0.543	0.023	0.0029	6.30E-15	FAM178A
rs7899004	10	104331425	T	C	0.558	0.025	0.0029	7.00E-17	SUFU
rs6584575	10	105567399	A	G	0.08	0.034	0.0052	9.50E-11	SH3PXD2A
rs291979	10	121119787	A	G	0.225	2.90E-02	0.0035	2.00E-16	GRK5
rs1614303	10	123386796	T	G	0.867	2.20E-02	0.0038	5.70E-09	FGFR2
rs10794175	10	126348063	T	G	0.296	2.00E-02	0.003	6.40E-12	FAM53B
rs12779328	10	12983979	T	C	0.35	-2.80E-02	0.0033	1.70E-17	CCDC3
rs4350272	10	25096124	A	G	0.367	0.02	0.0033	2.60E-09	ARHGAP21
rs7069985	10	27930837	A	G	0.808	-2.30E-02	0.0034	1.60E-11	RAB18
rs4332428	10	4955434	A	G	0.881	3.60E-02	0.0045	1.50E-15	AKR1C1
rs10995319	10	52432893	T	C	0.733	1.90E-02	0.0034	3.20E-08	PRKG1
rs1171615	10	61139096	T	C	0.833	-0.022	0.0038	5.80E-09	SLC16A9
rs10997979	10	69607198	A	G	0.433	-0.021	0.0029	3.80E-13	MYPN
rs2631676	10	93027389	A	G	0.725	-2.80E-02	0.0039	4.60E-13	PCGF5
rs10790381	11	119762705	A	G	0.792	0.027	0.0038	2.00E-12	ARHGEF12
rs1461503	11	122350285	A	C	0.45	-0.018	0.0029	4.90E-10	BSX
rs6485978	11	12634991	T	C	0.545	-2.30E-02	0.0029	1.40E-15	TEAD1
rs2272566	11	234552	A	G	0.373	1.60E-02	0.0029	2.60E-08	PSMD13
rs2237886	11	2767307	T	C	0.108	0.043	0.0049	5.30E-18	KCNQ1
rs10767838	11	30304503	A	G	0.767	0.025	0.0033	2.60E-14	C11orf46
rs3802758	11	45892611	A	G	0.966	3.90E-02	0.0066	2.20E-09	PEX16
rs1681630	11	47925728	T	C	0.242	2.90E-02	0.0031	2.40E-20	PTPRJ
rs3782089	11	65093395	T	C	0.025	-5.30E-02	0.0066	4.60E-16	SSSCA1
rs7112925	11	66582736	T	C	0.358	-0.024	0.003	6.40E-15	RHOD
rs606452	11	74953826	A	C	0.158	0.043	0.0043	1.90E-23	SERPINH1
rs2164747	12	102868966	A	G	0.892	-0.029	0.0049	4.70E-09	HSP90B1
rs2888893	12	105862761	T	C	0.602	-0.017	0.003	8.90E-09	C12orf23
rs11616067	12	114877557	A	G	0.75	2.10E-02	0.0035	8.00E-09	MED13L
rs2856321	12	11747040	A	G	0.603	-3.10E-02	0.003	7.60E-24	ETV6
rs11835818	12	120979192	T	C	0.558	-2.20E-02	0.0029	2.00E-13	BCL7A
rs7980687	12	122388664	A	G	0.183	0.039	0.0037	1.00E-26	SBNO1
rs10770705	12	20748734	A	C	0.339	3.00E-02	0.0031	2.30E-21	SLCO1C1
rs11049611	12	28491511	T	C	0.342	-3.80E-02	0.0032	2.50E-32	CCDC91
rs11612228	12	447245	T	C	0.308	2.00E-02	0.0032	6.50E-10	B4GALNT3
rs10880969	12	45113290	T	C	0.163	-2.40E-02	0.0033	6.20E-13	SLC38A2
rs2306694	12	54966903	A	G	0.908	-4.60E-02	0.0057	1.50E-15	CS
rs10877030	12	56542981	T	G	0.608	0.023	0.0031	4.30E-13	CTDSP2
rs17122659	12	58243190	A	G	0.892	-0.031	0.0049	5.20E-10	SLC16A7
rs8756	12	64646019	A	C	0.534	-5.90E-02	0.0029	4.50E-90	HMGA2
rs10748128	12	68113925	T	G	0.348	0.038	0.0034	4.40E-29	FRS2
rs17783015	12	88755517	T	C	0.208	-0.023	0.004	1.50E-08	ATP2B1
rs3825199	12	92501085	A	G	0.8	-5.10E-02	0.0035	3.90E-49	SOCS2
rs1199734	13	20468246	T	G	0.167	-2.20E-02	0.0039	1.60E-08	LATS2
rs11618507	13	29070751	T	G	0.275	2.30E-02	0.0036	3.40E-10	SLC7A1
rs12323101	13	32041406	A	G	0.333	2.10E-02	0.003	1.40E-11	PDS5B
rs6561319	13	46010121	A	C	0.633	0.021	0.0031	9.80E-12	LRCH1
rs3118905	13	50003335	A	G	0.237	-0.058	0.0033	1.10E-69	DLEU7
rs3818416	13	77372469	A	C	0.208	-2.10E-02	0.0035	1.70E-09	EDNRB
rs11616380	13	79603316	T	G	0.275	1.90E-02	0.0033	1.20E-08	SPRY2
rs7319045	13	90822575	A	G	0.39	0.024	0.003	8.40E-15	GPC5
rs8017130	14	22828996	A	G	0.358	-2.30E-02	0.0034	1.00E-11	HOMEZ
rs1950500	14	23900690	T	C	0.233	3.10E-02	0.0032	3.20E-22	NFATC4
rs12435366	14	34908140	T	C	0.254	-2.30E-02	0.0035	4.90E-11	NFKBIA
rs10131337	14	36214267	T	C	0.203	2.70E-02	0.0038	2.90E-12	PAX9
rs8006657	14	54314899	A	G	0.33	-0.022	0.003	6.60E-13	SAMD4A
rs11624136	14	58758573	A	G	0.45	1.80E-02	0.0029	1.90E-09	DAAM1
rs2093210	14	60027032	T	C	0.596	-0.039	0.0031	3.00E-35	C14orf39
rs2781373	14	64637968	A	G	0.347	-0.021	0.003	3.70E-12	MAX

rs1980850	14	67716941	A	G	0.192	-3.00E-02	0.004	3.80E-14	RAD51L1
rs2058092	14	73002719	T	C	0.61	1.70E-02	0.003	1.20E-08	NUMB
rs862034	14	74060499	A	G	0.49	-2.80E-02	0.003	6.40E-20	LTBP2
rs7154721	14	91497101	T	C	0.6	0.027	0.0029	4.60E-20	TRIP11
rs1036477	15	46702218	A	G	0.917	3.20E-02	0.0048	3.70E-11	FBN1
rs16964211	15	49317787	A	G	0.042	-5.70E-02	0.0071	1.20E-15	CYP19A1
rs7177711	15	60167263	A	G	0.517	0.021	0.0029	5.40E-13	FAM148A
rs7162825	15	61226239	T	C	0.517	0.016	0.0029	3.60E-08	LACTB
rs17264185	15	64784141	A	G	0.783	-2.00E-02	0.0033	1.10E-09	SMAD6
rs975210	15	68151406	A	G	0.167	3.50E-02	0.0041	1.40E-17	TLE3
rs12904334	15	70629759	A	G	0.008	8.40E-02	0.013	3.70E-11	ARIH1
rs5742915	15	72123686	T	C	0.45	-0.035	0.0031	1.50E-29	PML
rs11855014	15	83529838	A	G	0.246	-0.022	0.0034	9.20E-11	PDE8A
rs2280470	15	87196630	A	G	0.339	4.40E-02	0.0031	2.80E-44	ACAN
rs7181724	15	92352611	A	G	0.528	-2.00E-02	0.0032	1.00E-10	MCTP2
rs4548838	15	98578713	T	C	0.45	3.30E-02	0.003	8.60E-28	ADAMTS17
rs1659127	16	14295806	A	G	0.3	3.00E-02	0.0033	2.80E-19	MKL2
rs2023693	16	20787541	A	G	0.383	-1.70E-02	0.003	2.40E-08	DCUN1D3
rs11642612	16	29937696	A	C	0.559	-0.016	0.003	4.20E-08	FLJ25404
rs4785393	16	48816984	A	G	0.817	-2.30E-02	0.004	1.30E-08	PAPD5
rs8058684	16	52072619	A	G	0.308	2.10E-02	0.0032	1.20E-10	RBL2
rs217181	16	70671503	T	C	0.2	2.40E-02	0.0038	3.70E-10	HPR
rs11648796	16	732191	A	G	0.733	-3.30E-02	0.0038	1.40E-18	NARFL
rs11640018	16	73885809	T	C	0.638	-0.019	0.0032	2.30E-09	CFDP1
rs6420435	16	80741702	A	C	0.254	2.30E-02	0.0037	2.50E-10	MPHOSPH6
rs2326458	16	83545180	A	C	0.833	-2.20E-02	0.0035	5.00E-10	ZDHHC7
rs4843367	16	84975391	T	C	0.325	-1.90E-02	0.0032	1.80E-09	FOXF1
rs8052560	16	87304743	A	C	0.783	0.036	0.0044	3.80E-16	C16orf84
rs4640244	17	21224816	A	G	0.622	2.50E-02	0.0034	1.50E-13	KCNJ12
rs3809790	17	24979666	T	C	0.475	-1.60E-02	0.0029	4.80E-08	SSH2
rs3760318	17	26271841	A	G	0.358	-4.10E-02	0.003	3.00E-41	CENTA2
rs2338115	17	34183104	T	C	0.558	2.30E-02	0.0029	1.10E-14	PIP4K2B
rs584828	17	35852756	T	C	0.433	-0.025	0.003	3.50E-17	IGFBP4
rs9766	17	38106367	A	G	0.553	2.10E-02	0.0029	9.00E-13	EZH1
rs4986172	17	40571807	T	C	0.325	-3.40E-02	0.0032	7.60E-27	ACBD4
rs318095	17	44329733	T	C	0.517	0.024	0.0029	1.50E-16	ATP5G1
rs870183	17	546561	A	G	0.475	-1.70E-02	0.0029	6.20E-09	VPS53
rs2079795	17	56851431	T	C	0.3	4.50E-02	0.0031	1.70E-46	C17orf82
rs3923086	17	60979950	A	C	0.392	-0.024	0.0033	4.70E-13	AXIN2
rs2072268	17	63814947	A	G	0.525	-2.00E-02	0.0031	1.10E-10	ARSG
rs11867479	17	65601802	T	C	0.325	0.026	0.0032	2.20E-16	KCNJ16
rs10083886	17	67434950	T	C	0.217	1.90E-02	0.0033	6.60E-09	SOX9
rs2117563	17	70880580	A	G	0.134	-2.40E-02	0.0039	1.20E-09	GRB2
rs9217	17	7303812	T	C	0.608	-2.80E-02	0.003	4.60E-20	ZBTB4
rs1552173	17	74230437	T	C	0.542	-0.018	0.0029	7.90E-10	PSCD1
rs4239020	17	77769930	T	C	0.708	-0.021	0.0031	8.80E-12	CCDC57
rs692964	18	13084132	A	G	0.558	-1.90E-02	0.003	1.90E-10	CEP192
rs14062	18	17704301	A	G	0.342	-1.80E-02	0.0031	7.80E-09	MIB1
rs4369779	18	18989406	T	C	0.258	-5.60E-02	0.0036	1.50E-53	CABLES1
rs888403	18	2756938	A	G	0.633	-1.90E-02	0.0033	9.30E-09	SMCHD1
rs11152213	18	56003928	A	C	0.717	-2.50E-02	0.0035	6.90E-13	MC4R
rs8097893	18	73112043	A	G	0.933	4.20E-02	0.0068	4.60E-10	GALR1
rs11659752	18	75323850	T	G	0.75	0.024	0.0034	5.90E-13	NFATC1
rs8102380	19	10662185	A	G	0.633	-0.02	0.0031	8.00E-11	ILF3
rs7259684	19	12047611	A	G	0.888	-3.50E-02	0.0064	4.10E-08	LOC729747
rs8103068	19	17383869	T	C	0.867	3.10E-02	0.0046	1.50E-11	BST2
rs8103992	19	19526643	A	C	0.22	0.029	0.0037	1.20E-14	PBX4
rs11880992	19	2127403	A	G	0.375	0.033	0.003	6.90E-28	DOT1L
rs2074977	19	3385028	A	C	0.638	-0.029	0.0031	1.90E-20	NFIC
rs7253628	19	35739109	A	G	0.833	-2.40E-02	0.0039	1.50E-09	ZNF536
rs4802134	19	43038525	A	G	0.193	2.70E-02	0.004	2.60E-11	SIPA1L3

rs4803468	19	46614192	A	G	0.398	0.03	0.0031	1.70E-21	BCKDHA
rs2682587	19	48774269	A	C	0.167	2.30E-02	0.0038	2.00E-09	XRCC1
rs2123731	19	4880473	A	G	0.693	2.30E-02	0.0035	2.00E-11	UHRF1
rs891088	19	7135762	A	G	0.728	-2.90E-02	0.0034	7.20E-18	INSR
rs7517682	1	103292177	A	G	0.585	-0.023	0.003	3.60E-14	COL11A1
rs10779751	1	11206923	A	G	0.292	0.021	0.0032	2.00E-10	FRAP1
rs12120956	1	113004094	A	G	0.172	-2.50E-02	0.0035	2.10E-12	CAPZA1
rs9428104	1	118657110	A	G	0.314	-4.30E-02	0.0034	2.80E-36	SPAG17
rs6658763	1	145158997	T	C	0.125	-0.036	0.0054	3.60E-11	FMO5
rs2298265	1	149525667	T	C	0.117	-0.03	0.0046	8.30E-11	ZNF687
rs6688100	1	158666210	T	C	0.466	1.60E-02	0.0029	2.30E-08	VANGL2
rs4656220	1	168915901	T	C	0.466	2.10E-02	0.0032	1.50E-10	PRRX1
rs6694089	1	170350504	A	G	0.217	0.039	0.0032	4.20E-33	DNM3
rs1325596	1	175060689	A	G	0.508	0.025	0.0029	9.70E-18	PAPPA2
rs3814333	1	182273742	T	C	0.342	4.90E-02	0.0032	4.80E-51	GLT25D2
rs12137162	1	19635983	A	C	0.271	0.019	0.0032	4.10E-09	CAPZB
rs425277	1	2059032	T	C	0.28	2.80E-02	0.0033	1.20E-17	PRKCZ
rs10863936	1	210304421	A	G	0.55	-2.00E-02	0.0029	1.10E-11	DTL
rs6540834	1	212694042	T	C	0.384	-2.70E-02	0.0033	2.60E-16	PTPN14
rs212524	1	21455898	T	C	0.433	-0.021	0.003	5.40E-12	ECE1
rs991967	1	216682074	A	C	0.81	-0.034	0.0032	1.80E-26	TGFB2
rs6696239	1	225816691	A	G	0.233	-3.80E-02	0.0037	7.10E-24	ZNF678
rs2806561	1	23377382	A	G	0.567	2.70E-02	0.0029	1.90E-20	LUZP1
rs11799609	1	241684940	T	G	0.142	2.60E-02	0.0042	1.10E-09	SDCCAG8
rs4601530	1	24916698	T	C	0.25	-2.50E-02	0.0033	2.70E-14	CLIC4
rs16834765	1	32144029	T	C	0.058	0.045	0.0064	1.50E-12	PTP4A2
rs7544462	1	37735343	A	C	0.925	3.20E-02	0.0053	1.50E-09	C1orf149
rs6600365	1	41328840	T	C	0.6	-2.70E-02	0.0029	1.70E-20	SCMH1
rs3014219	1	45797041	A	G	0.4	-2.10E-02	0.0029	9.90E-13	AKR1A1
rs12855	1	51212681	T	C	0.108	0.038	0.0051	7.30E-14	CDKN2C
rs6691924	1	54726833	T	C	0.85	3.20E-02	0.005	2.40E-10	ACOT11
rs2815379	1	67283062	A	G	0.242	-1.80E-02	0.0033	1.60E-08	SLC35D1
rs17391694	1	78396214	T	C	0.136	4.30E-02	0.0053	3.90E-16	GIPC2
rs9434723	1	9214869	A	G	0.117	0.029	0.0041	9.10E-13	H6PD
rs2811594	1	93115870	A	G	0.333	-2.40E-02	0.0032	4.10E-14	FAM69A
rs17113369	1	95559811	T	C	0.992	7.30E-02	0.013	5.90E-09	RWDD3
rs6080830	20	17719113	A	G	0.575	1.60E-02	0.0029	4.20E-08	BANF2
rs143384	20	33489170	A	G	0.6	-0.075	0.0032	1.10E-121	GDF5
rs4812586	20	34978087	A	G	0.788	3.00E-02	0.004	8.60E-14	SAMHD1
rs2224538	20	37985492	T	C	0.644	0.017	0.0031	1.90E-08	MAFB
rs7273787	20	4046567	A	G	0.667	-2.20E-02	0.0031	2.80E-12	SMOX
rs1326023	20	54275785	A	G	0.325	2.40E-02	0.0032	1.10E-13	MC3R
rs2057291	20	56905438	A	G	0.35	0.02	0.0032	2.20E-10	GNAS
rs6061231	20	60390312	A	C	0.217	-2.10E-02	0.0032	1.00E-10	RPS21
rs1884897	20	6560832	A	G	0.375	4.40E-02	0.003	1.30E-48	BMP2
rs2829941	21	26130806	T	G	0.608	1.70E-02	0.003	3.30E-08	APP
rs2211866	21	38609977	A	G	0.458	2.20E-02	0.003	3.50E-13	KCNJ15
rs9977276	21	46260755	T	G	0.25	-2.20E-02	0.0035	2.90E-10	COL6A1
rs7284476	22	36459278	A	G	0.474	1.80E-02	0.003	6.10E-09	TRIOBP
rs738288	22	38237607	A	G	0.442	-2.00E-02	0.003	5.50E-11	SMCR7L
rs3885668	2	10095930	T	C	0.558	-0.022	0.003	8.40E-13	KLF11
rs13388725	2	108413622	A	G	0.617	-1.80E-02	0.003	2.00E-09	GCC2
rs2166898	2	121329129	A	G	0.169	-0.027	0.0041	8.60E-11	GLI2
rs7567288	2	134151294	T	C	0.81	-2.90E-02	0.0038	3.00E-14	NAP5
rs749234	2	144947819	A	G	0.317	1.70E-02	0.0031	2.30E-08	ZEB2
rs17038954	2	1624680	T	C	0.042	4.40E-02	0.0062	1.30E-12	PXDN
rs540652	2	169415674	T	C	0.492	2.10E-02	0.0029	3.00E-12	NOSTRIN
rs12987566	2	171860892	T	C	0.225	2.40E-02	0.0033	1.20E-12	METTL8
rs6746356	2	174524144	A	C	0.7	0.019	0.0034	1.20E-08	SP3
rs833152	2	182927346	A	C	0.483	-1.70E-02	0.003	2.10E-08	PDE1A
rs2345835	2	18438433	T	C	0.483	-1.80E-02	0.003	2.20E-09	RDH14

rs12693589	2	191540907	T	C	0.754	-2.20E-02	0.0034	9.10E-11	STAT1
rs6435143	2	202902501	A	C	0.442	1.90E-02	0.003	1.50E-10	NOP5/NOP58
rs12470505	2	219616613	T	G	0.842	0.048	0.0049	6.20E-22	CCDC108
rs6761041	2	224738373	T	C	0.556	2.30E-02	0.0029	3.00E-15	SERPINE2
rs3116168	2	232698075	T	C	0.325	-3.90E-02	0.0033	1.40E-31	DIS3L2
rs2289195	2	25316987	A	G	0.367	3.80E-02	0.003	2.40E-37	DNMT3A
rs780094	2	27594741	T	C	0.383	-0.021	0.003	5.70E-12	GCKR
rs6714546	2	33214929	A	G	0.274	-3.00E-02	0.0034	1.60E-18	LTBP1
rs13416119	2	42316434	A	G	0.925	0.029	0.005	1.50E-08	EML4
rs9309101	2	43483116	A	G	0.653	-2.10E-02	0.0031	6.10E-11	THADA
rs897080	2	44627706	T	C	0.746	-0.028	0.0034	1.60E-16	C2orf34
rs12474201	2	46774789	A	G	0.317	2.80E-02	0.0031	2.30E-19	SOCS5
rs354196	2	54819911	A	G	0.449	-2.10E-02	0.003	5.60E-12	SPTBN1
rs3791679	2	55950396	A	G	0.72	6.00E-02	0.0035	2.40E-67	EFEMP1
rs2120335	2	68348506	A	G	0.342	-1.90E-02	0.003	8.40E-10	PPP3R1
rs7568069	2	71437993	A	G	0.667	-2.20E-02	0.0029	2.60E-13	ZNF638
rs11684404	2	88705737	T	C	0.7	-0.032	0.0031	9.00E-25	EIF2AK3
rs6439168	3	130533633	A	G	0.183	-0.037	0.0036	7.70E-25	H1FX
rs2597513	3	13530836	T	C	0.875	-3.90E-02	0.0048	3.10E-16	HDAC11
rs9880211	3	137590239	A	G	0.142	-0.03	0.0034	1.60E-18	STAG1
rs724016	3	142588260	A	G	0.517	-7.80E-02	0.0029	3.20E-158	ZBTB38
rs936339	3	144018195	T	C	0.175	0.022	0.0039	1.80E-08	PCOLCE2
rs6441170	3	159289654	T	C	0.638	-2.20E-02	0.003	9.70E-13	SHOX2
rs9858528	3	184838099	A	G	0.758	2.20E-02	0.0033	3.50E-11	KLHL24
rs720390	3	187031377	A	G	0.383	3.50E-02	0.0031	1.20E-29	IGF2BP2
rs4686904	3	188921216	T	C	0.642	-2.10E-02	0.0031	2.50E-11	BCL6
rs9841435	3	192593854	A	G	0.617	-2.00E-02	0.0031	2.40E-10	CCDC50
rs3915129	3	41218746	T	G	0.483	-1.60E-02	0.0029	4.00E-08	CTNNB1
rs2633761	3	4703104	A	G	0.583	1.60E-02	0.0029	2.60E-08	ITPR1
rs13088462	3	51046753	T	C	0.942	-5.90E-02	0.0068	7.90E-18	DOCK3
rs2581830	3	53109138	T	C	0.358	3.10E-02	0.003	4.40E-25	RFT1
rs2034172	3	55386803	A	G	0.383	-1.80E-02	0.0033	4.10E-08	WNT5A
rs1658351	3	57988613	T	C	0.678	-2.30E-02	0.0031	9.90E-13	FLNB
rs6794009	3	61488535	A	G	0.508	-0.016	0.0029	3.90E-08	PTPRG
rs17806888	3	67499012	T	C	0.908	0.034	0.0048	2.90E-12	SUCLG2
rs2175513	3	68705056	A	G	0.576	-0.017	0.003	3.00E-08	FAM19A1
rs12330322	3	72538045	T	C	0.225	-3.40E-02	0.0035	3.30E-22	RYBP
rs12639764	4	106435654	T	C	0.543	2.70E-02	0.003	1.60E-19	TET2
rs1562975	4	109628057	A	G	0.297	2.50E-02	0.0032	5.50E-15	RPL34
rs7659107	4	114961698	A	G	0.742	-2.40E-02	0.0035	9.10E-12	CAMK2D
rs6838153	4	122940449	A	G	0.667	-2.20E-02	0.0031	2.60E-12	EXOSC9
rs12513181	4	124055106	A	C	0.728	-0.02	0.0033	3.20E-09	NUDT6
rs763318	4	12572672	A	G	0.576	-2.10E-02	0.0029	8.00E-13	RAB28
rs1812175	4	145794294	A	G	0.192	-7.90E-02	0.004	2.10E-86	HHIP
rs13150868	4	152400121	T	G	0.527	1.70E-02	0.003	2.20E-08	ESSPL
rs3958122	4	1663729	T	C	0.449	2.70E-02	0.0031	2.60E-18	SLBP
rs955748	4	184452669	A	G	0.319	-2.80E-02	0.0034	3.10E-16	WWC2
rs2306596	4	39020335	A	C	0.492	1.90E-02	0.003	7.80E-11	RFC1
rs1996422	4	48382108	A	G	0.717	-0.022	0.0033	2.80E-11	FRYL
rs6446315	4	5086488	A	G	0.867	-2.80E-02	0.0043	4.20E-11	CYTL1
rs13113518	4	56094405	T	C	0.625	-1.80E-02	0.003	8.10E-09	CLOCK
rs17081935	4	57518233	T	C	0.169	3.10E-02	0.0037	6.70E-17	C4orf14
rs9993613	4	73694878	T	G	0.509	0.03	0.0029	4.50E-24	ADAMTS3
rs17556750	4	82374592	A	C	0.275	0.046	0.0032	8.30E-48	PRKG2
rs2302580	4	8659534	T	C	0.491	-2.90E-02	0.0036	4.20E-15	CPZ
rs13177718	5	108141243	T	C	0.071	-4.30E-02	0.0059	2.90E-13	FER
rs1582931	5	122685098	A	G	0.458	-2.80E-02	0.003	2.60E-20	CCDC100
rs26024	5	127723921	A	C	0.617	-2.30E-02	0.0031	3.20E-14	FBN2
rs7701414	5	131613857	A	G	0.517	-3.70E-02	0.003	1.30E-34	PDLIM4
rs526896	5	134384604	T	G	0.683	3.60E-02	0.0034	2.30E-25	PITX1
rs165189	5	139125931	A	G	0.88	-2.90E-02	0.0046	1.60E-10	PSD2

rs4624820	5	141661972	A	G	0.4	1.80E-02	0.0029	1.00E-09	SPRY4
rs2974438	5	168183481	A	G	0.183	-0.037	0.0036	4.00E-24	SLIT3
rs4868126	5	171216074	T	G	0.35	-0.036	0.0032	2.80E-29	FBXW11
rs7733195	5	172927230	A	G	0.375	-2.90E-02	0.003	2.70E-21	FAM44B
rs422421	5	176449932	T	C	0.22	-0.034	0.0036	1.80E-20	FGFR4
rs11750568	5	178468319	A	G	0.433	2.00E-02	0.0031	6.20E-11	ADAMTS2
rs6879260	5	179663620	T	C	0.342	-2.70E-02	0.0031	1.90E-18	GFPT2
rs17410035	5	31576899	T	G	0.264	1.90E-02	0.0031	9.00E-10	C5orf22
rs9292468	5	32854830	T	C	0.475	0.036	0.003	1.50E-33	C5orf23
rs301901	5	37082383	A	G	0.633	0.024	0.0029	4.30E-16	NIPBL
rs3812040	5	39461777	T	C	0.714	2.40E-02	0.0033	2.40E-13	DAB2
rs17574650	5	42472673	A	C	0.908	-0.038	0.0054	2.10E-12	GHR
rs7716219	5	54990828	T	C	0.292	3.00E-02	0.0031	7.20E-22	SLC38A9
rs2662027	5	56290242	T	G	0.083	-3.30E-02	0.0048	6.30E-12	MIER3
rs7727731	5	64710202	T	C	0.083	3.30E-02	0.0049	1.30E-11	ADAMTS6
rs9291926	5	67635412	T	G	0.483	0.019	0.0031	3.40E-10	PIK3R1
rs34651	5	72179761	T	C	0.905	-4.10E-02	0.0058	2.20E-12	TNPO1
rs820848	5	74000416	A	G	0.708	-2.10E-02	0.0035	3.30E-09	HEXB
rs12519505	5	77541632	T	C	0.192	-0.022	0.0036	8.50E-10	AP3B1
rs32855	5	79871948	A	G	0.783	2.40E-02	0.0036	5.80E-11	FAM151B
rs6894139	5	88363538	T	G	0.534	3.00E-02	0.003	6.10E-24	MEF2C
rs314263	6	105499438	T	C	0.627	-4.30E-02	0.0031	1.00E-42	LIN28B
rs6920372	6	109830632	A	G	0.364	-2.50E-02	0.0029	1.70E-17	PPIL6
rs2145357	6	116558135	A	G	0.708	-2.10E-02	0.0033	3.90E-10	NT5DC1
rs1405212	6	117597357	T	C	0.355	-2.30E-02	0.003	1.80E-14	VGLL2
rs1155939	6	126907826	A	C	0.433	4.20E-02	0.0029	9.60E-46	C6orf173
rs4896582	6	142745570	A	G	0.267	-5.10E-02	0.0032	2.60E-55	GPR126
rs6902771	6	152199574	T	C	0.417	0.031	0.003	7.20E-25	ESR1
rs11156098	6	156629523	T	C	0.043	2.70E-02	0.0047	1.80E-08	ARID1B
rs1832871	6	158642022	A	G	0.367	2.50E-02	0.0031	1.80E-15	TULP4
rs991946	6	166249852	T	C	0.5	-0.021	0.0029	8.40E-13	T
rs2763273	6	168577472	T	C	0.241	-2.20E-02	0.0034	2.80E-10	SMOC2
rs17330192	6	17697354	T	C	0.8	-1.90E-02	0.0033	1.80E-08	FAM8A1
rs1047014	6	19949472	T	C	0.725	-3.20E-02	0.0036	1.30E-18	ID4
rs932445	6	2112224	T	C	0.575	0.018	0.003	6.00E-09	GMDS
rs806794	6	26308656	A	G	0.724	0.06	0.0033	4.60E-74	HIST1H2BF
rs1233627	6	28859706	T	C	0.5	2.40E-02	0.0029	3.10E-16	TRIM27
rs9404952	6	29912144	A	G	0.483	0.018	0.003	2.50E-09	HLA-G
rs6457374	6	31380240	T	C	0.608	-4.10E-02	0.0034	8.30E-35	HLA-C
rs12214804	6	34296844	T	C	0.925	-8.40E-02	0.0057	1.50E-49	HMGA1
rs16895130	6	42032909	A	G	0.636	-0.023	0.0033	4.30E-12	CCND3
rs10948222	6	45352393	T	C	0.387	-3.10E-02	0.0033	9.80E-21	SUPT3H
rs9395264	6	47582981	T	G	0.342	-2.00E-02	0.0031	2.00E-10	CD2AP
rs12209223	6	76221309	A	C	0.129	5.10E-02	0.0049	4.90E-25	FILIP1
rs9392918	6	7653630	T	C	0.5	-0.038	0.0029	3.80E-38	BMP6
rs310421	6	81848782	T	G	0.483	3.20E-02	0.0029	3.30E-27	FAM46A
rs761391	6	85504822	T	C	0.569	-0.019	0.0034	8.30E-09	TBX18
rs6952113	7	120564855	A	G	0.375	-1.80E-02	0.003	1.20E-09	C7orf58
rs929637	7	12243047	T	G	0.207	-2.10E-02	0.0035	2.00E-09	TMEM106B
rs6962887	7	134696326	T	G	0.655	2.30E-02	0.0034	6.10E-11	CNOT4
rs273945	7	137262106	A	C	0.466	-1.90E-02	0.0031	1.40E-09	CREB3L2
rs822531	7	148260692	T	C	0.764	3.60E-02	0.004	1.70E-18	EZH2
rs6955948	7	150139653	T	C	0.263	0.031	0.0034	5.20E-20	TMEM176A
rs3807931	7	20348199	A	G	0.408	2.70E-02	0.0029	1.20E-19	ITGB8
rs12538407	7	23487841	A	G	0.617	0.032	0.003	3.50E-26	IGF2BP3
rs1055144	7	25837634	T	C	0.183	0.021	0.0037	7.80E-09	NFE2L3
rs798497	7	2762483	A	G	0.712	5.70E-02	0.0032	2.20E-71	GNA12
rs552707	7	28171828	T	C	0.314	4.60E-02	0.0032	9.30E-46	JAZF1
rs6462432	7	32902049	A	G	0.298	0.017	0.003	2.60E-08	KBTBD2
rs2715094	7	50697946	A	G	0.75	-0.021	0.0034	9.20E-10	GRB10
rs1113765	7	55856828	A	G	0.192	-2.40E-02	0.0038	1.70E-10	41883

rs12669267	7	72942572	T	C	0.135	-2.90E-02	0.0052	3.00E-08	WBSCR28
rs17807185	7	77146231	A	G	0.592	-2.20E-02	0.003	3.90E-13	RSBN1L
rs4725061	7	8053164	A	G	0.578	-0.02	0.0031	1.10E-10	GLCCI1
rs42039	7	92082358	T	C	0.283	0.068	0.0034	3.80E-88	CDK6
rs17250196	7	99655132	T	G	0.061	0.045	0.0072	4.20E-10	GATS/PVRIG
rs1550162	8	117632713	A	G	0.725	-2.40E-02	0.0032	1.30E-13	EIF3H
rs1599473	8	120544539	T	G	0.15	-2.70E-02	0.0034	1.00E-14	NOV
rs4733724	8	130792910	A	G	0.808	0.05	0.0037	1.40E-41	MLZE
rs7834383	8	13317848	T	G	0.397	2.20E-02	0.0032	1.50E-11	DLC1
rs1036821	8	135719665	A	G	0.317	-3.70E-02	0.0032	1.10E-30	ZFAT
rs2013265	8	24148445	T	C	0.242	-0.028	0.0033	2.40E-16	ADAM28
rs568610	8	27583914	T	C	0.208	2.20E-02	0.0034	1.40E-10	SCARA3
rs6988484	8	49576333	T	C	0.683	-0.022	0.0033	4.80E-11	EFCAB1
rs9650315	8	57318152	T	G	0.125	-0.061	0.0045	1.50E-41	CHCHD7
rs2956605	8	76045609	A	C	0.383	2.40E-02	0.0031	5.10E-15	CRISPLD1
rs429433	8	8785304	A	G	0.05	0.046	0.0071	1.30E-10	MFHAS1
rs989393	9	100783157	T	C	0.7	2.20E-02	0.0032	3.30E-11	COL15A1
rs7027110	9	108638867	A	G	0.258	3.20E-02	0.0034	2.10E-20	ZNF462
rs3739707	9	112832527	A	C	0.3	-2.40E-02	0.0035	4.00E-12	LPAR1
rs7033487	9	118169078	T	C	0.775	3.70E-02	0.0036	1.10E-24	PAPPA
rs7466269	9	132453905	A	G	0.667	3.30E-02	0.0031	1.00E-27	FUBP3
rs3132297	9	136441687	A	G	0.147	-2.30E-02	0.0042	3.80E-08	RXRA
rs7849585	9	138251691	T	G	0.317	3.60E-02	0.0032	1.10E-29	QSOX2
rs1576900	9	18619792	A	G	0.28	-0.019	0.0033	6.60E-09	ADAMTSL1
rs11144688	9	77732106	A	G	0.095	-6.30E-02	0.0063	5.70E-24	PCSK5
rs7853235	9	85850602	T	C	0.15	0.029	0.0037	7.50E-15	RMI1
rs181338	9	88297981	T	C	0.492	2.80E-02	0.0029	2.50E-22	ZCCHC6
rs7043114	9	94427804	T	C	0.517	-2.90E-02	0.0029	1.80E-22	IPPK
rs817300	9	97420043	A	G	0.035	-0.085	0.0069	4.30E-34	PTCH1

Supplementary Table 3c: Summary of the systolic blood pressure SNPs previously identified as associated with systolic blood pressure at genome wide significance

SNP	Chr	Position (bp	A1	A2	Frequency A1	Beta	SE	P	Nearest Gene
rs17367504	1	11785365	G	A	0.15	-0.861270375	0.135569	2.11E-10	MTHFR-NPPB
rs2932538	1	113018066	G	A	0.75	0.538172767	0.114735	2.72E-06	MOV10
rs13082711	3	27512913	T	C	0.78	-0.308571186	0.118198	0.009038	SLC4A7
rs419076	3	170583580	T	C	0.47	0.519099789	0.096703	7.96E-08	MECOM
rs13107325	4	103407732	T	C	0.05	-1.07405989	0.208449	2.57E-07	SLC39A8
rs1458038	4	81383747	T	C	0.29	0.662369723	0.110612	2.12E-09	FGF5
rs1173771	5	32850785	G	A	0.60	0.518884302	0.098626	1.43E-07	NPR3-C5orf23
rs805303	6	31724345	G	A	0.61	0.482412818	0.099617	1.28E-06	BAT2-BAT5
rs11191548	10	104836168	T	C	0.91	1.082956457	0.174159	5.03E-10	CYP17A1-NT5C2
rs1813353	10	18747454	T	C	0.68	0.515202031	0.105114	9.52E-07	CACNB2(3')
rs932764	10	95885930	G	A	0.44	0.503919963	0.097615	2.44E-07	PLCE1
rs381815	11	16858844	T	C	0.26	0.65466	0.109758	2.45E-09	PLEKHA7
rs7129220	11	10307114	G	A	0.89	-0.823718311	0.155517	1.18E-07	ADM
rs17249754	12	88584717	G	A	0.84	0.955148491	0.133881	9.73E-13	ATP2B1
rs3184504	12	110368991	T	C	0.47	0.598422185	0.099323	1.69E-09	SH2B3
rs1378942	15	72864420	C	A	0.35	0.631582192	0.100605	3.43E-10	CYP1A1-ULK3
rs12940887	17	44757806	T	C	0.38	0.394575927	0.100154	8.16E-05	ZNF652
rs17608766	17	42368270	T	C	0.86	-0.705233925	0.142405	7.33E-07	GOSR2
rs1327235	20	10917030	G	A	0.46	0.358044305	0.097107	0.000227	JAG1
rs6015450	20	57184512	G	A	0.12	0.801129685	0.150225	9.67E-08	GNAS-EDN3

Supplementary Table 3d: Summary of the coronary artery disease SNPs previously identified as associated with coronary artery disease at genome wide significance

SNP	Chr	Position (bp)	A1	A2	Frequency A1	OR	CI95_low	CI95_up	Nearest gene
rs1122608	19	11163601	G	T	0.77	1.14	1.09	1.18	LDLR
rs11556924	7	129663496	C	T	0.62	1.09	1.07	1.12	ZC3HC1
rs12413409	10	104719096	G	A	0.89	1.12	1.08	1.16	CYP17A1, CNNM2, NT5C2
rs12936587	17	17543722	G	A	0.56	1.07	1.05	1.09	RASD1, SMCR3, PEMT
rs17114036	1	56962821	A	G	0.91	1.17	1.13	1.22	PPAP2B
rs1746048	10	44775824	C	T	0.87	1.09	1.07	1.13	CXCL12
rs17465637	1	222823529	C	A	0.74	1.14	1.09	1.2	MIA3
rs2306374	3	138119952	C	T	0.18	1.12	1.07	1.16	MRAS
rs2895811	14	100133942	C	T	0.43	1.07	1.05	1.1	HHIPL1
rs3798220	6	160961137	C	T	0.02	1.51	1.33	1.7	LPA
rs3825807	15	79089111	A	G	0.57	1.08	1.06	1.1	ADAMTS7
rs46522	17	46988597	T	C	0.53	1.06	1.04	1.08	UBE2Z, GIP, ATP5G1, SNF8
rs4773144	13	110960712	G	A	0.44	1.07	1.05	1.09	COL4A1, COL4A2
rs4977574	9	22098574	G	A	0.46	1.29	1.23	1.36	CDKN2A
rs579459	9	136154168	C	T	0.21	1.1	1.07	1.13	ABO
rs599839	1	109822166	A	G	0.78	1.11	1.08	1.15	SORT1
rs6725887	2	203745885	C	T	0.15	1.14	1.09	1.19	WDR12
rs9982601	21	35599128	T	C	0.15	1.18	1.12	1.24	MRPS6

Supplementary Table 3e: Summary of the type 2 diabetes SNPs previously identified as associated with type 2 diabetes at genome wide significance

SNP	Chr	Position (bp)	A1	A2	Frequency A1	OR	CI95_low	CI95_up	Nearest gene
rs4402960	3	186994381	T	G	[0.27-0.33]	1.13	1.09	1.17	IGF2BP2
rs7756992	6	20787688	G	A	[0.23-0.34]	1.2	1.16	1.25	CDKAL1
rs849135	7	28162938	G	A	[0.50-0.53]	1.12	1.08	1.17	JAZF1
rs3802177	8	118254206	G	A	[0.60-0.91]	1.16	1.11	1.22	SLC30A8
rs10811661	9	22124094	T	C	[0.81-0.84]	1.18	1.13	1.24	CDKN2A/B
rs1111875	10	94452862	C	T	[0.53-0.63]	1.15	1.11	1.19	HHEX/IDE
rs7903146	10	114748339	T	C	[0.18-0.38]	1.4	1.35	1.46	TCF7L2
rs9936385	16	52376670	C	T	[0.36-0.41]	1.13	1.09	1.18	FTO

Supplementary Table 3f: Summary of the educational attainment SNPs previously identified as associated with educational attainment at genome wide significance

SNP	Chr	Position (bp	A1	A2	Frequency	Beta	SE	P	Nearest Gene
rs13402908	2	100333377	T	C	0.5056	-0.018	0.003	1.70E-11	AFF3
rs4851251	2	100753490	T	C	0.2537	-0.017	0.003	1.91E-08	AFF3
rs1402025	5	113987898	T	C	0.7481	0.017	0.003	3.42E-08	AK097686
rs17824247	2	144152539	T	C	0.5802	-0.016	0.003	2.77E-09	ARHGAP15
rs3101246	4	42649935	T	G	0.5821	-0.015	0.003	1.43E-08	ATP8A1
rs2837992	21	42620520	T	C	0.3881	0.015	0.003	3.80E-08	BACE2
rs2457660	2	60757419	T	C	0.6063	-0.017	0.003	7.11E-10	BCL11A
rs11191193	10	103802408	A	G	0.6511	0.018	0.003	5.44E-11	C10orf76
rs62263923	3	85674790	A	G	0.6437	-0.016	0.003	7.01E-09	CADM2
rs113520408	7	128402782	A	G	0.2668	0.017	0.003	1.97E-08	CALU
rs35761247	3	48623124	A	G	0.05597	0.034	0.006	3.82E-08	COL7A1
rs17167170	7	133302345	A	G	0.7873	0.02	0.003	1.14E-09	EXOC4
rs12772375	10	104082688	T	G	0.416	-0.015	0.003	1.56E-08	GBF1
rs2615691	7	23402104	A	G	0.02799	-0.037	0.007	4.71E-08	IGF2BP3
rs2456973	12	56416928	A	C	0.6791	-0.02	0.003	1.06E-12	IKZF4
rs10061788	5	87934707	A	G	0.2164	0.021	0.004	2.46E-09	LINC00461
rs324886	5	87896602	T	C	0.3601	-0.015	0.003	1.91E-08	LINC00461
rs4863692	4	140764124	T	G	0.334	0.018	0.003	1.56E-10	MAML3
rs11588857	1	204587047	A	G	0.209	0.02	0.003	5.27E-10	MDM4
rs12646808	4	3249828	T	C	0.6418	0.016	0.003	4.00E-08	MSANTD1
rs165633	22	29880773	A	G	0.7948	-0.018	0.003	2.86E-09	NEFH
rs34305371	1	72733610	A	G	0.08769	0.035	0.005	3.76E-14	NEGR1
rs76076331	2	10977585	T	C	0.09328	0.02	0.004	3.63E-08	PDIA6
rs12531458	7	39090698	A	C	0.5354	0.014	0.003	3.11E-08	POU6F2
rs1043209	14	23373986	A	G	0.6026	0.018	0.003	1.82E-11	RBM23
rs112634398	3	50075494	A	G	0.95896	0.036	0.007	4.61E-08	RBM6
rs301800	1	8490603	T	C	0.1791	0.019	0.003	1.79E-08	RERE
rs7767938	6	153367613	T	C	0.7705	0.017	0.003	2.44E-08	RGS17
rs148734725	3	49406708	A	G	0.3078	0.025	0.003	1.36E-18	RHOA
rs4500960	2	162818621	T	C	0.4776	-0.016	0.003	3.75E-10	SLC4A10
rs572016	12	121279083	A	G	0.4907	0.014	0.003	3.46E-08	SPPL3
rs7945718	11	12748819	A	G	0.5951	0.015	0.003	1.54E-08	TEAD1
rs7854982	9	124644562	T	C	0.4869	-0.015	0.003	1.29E-08	TTLL11
rs10496091	2	61482261	A	G	0.2705	-0.018	0.003	5.62E-10	USP34
rs12987662	2	100821548	A	C	0.3787	0.027	0.003	2.69E-24	AFF3
rs9320913	6	98584733	A	C	0.5019	0.024	0.003	2.46E-19	AK091365
rs11712056	3	49914397	T	C	0.5504	0.024	0.003	3.30E-19	CAMKV
rs13294439	9	23358875	A	C	0.5765	-0.023	0.003	2.20E-17	CR627240
rs9537821	13	58402771	A	G	0.7631	0.024	0.003	1.50E-16	PCDH17
rs11210860	1	43982527	A	G	0.3694	0.017	0.003	2.36E-10	PTPRF
rs17119973	14	84913111	A	G	0.3078	-0.019	0.003	3.55E-10	FLRT2
rs1871109	9	1746016	T	G	0.5616	-0.016	0.003	4.35E-10	SMARCA2
rs1008078	1	91189731	T	C	0.3731	-0.016	0.003	6.01E-10	BARHL2
rs12671937	7	92654365	A	G	0.5634	0.016	0.003	9.15E-10	SAMD9
rs11768238	7	135227513	A	G	0.3246	-0.017	0.003	9.90E-10	NUP205
rs56231335	6	98187291	T	C	0.6474	-0.017	0.003	2.07E-09	AK091365
rs62259535	3	48939052	A	G	0.97015	0.048	0.008	2.63E-09	SLC25A20
rs16845580	2	161920884	T	C	0.6306	0.016	0.003	2.65E-09	TANK
rs2245901	2	194296294	A	G	0.4216	-0.016	0.003	4.54E-09	PCGEM1
rs2431108	5	103947968	T	C	0.6325	0.016	0.003	5.27E-09	NUDT12
rs12969294	18	35186122	A	G	0.3787	-0.016	0.003	7.24E-09	CELF4
rs1777827	1	211613114	A	G	0.569	0.015	0.003	1.55E-08	LINC00467
rs2568955	1	72762169	T	C	0.2369	-0.017	0.003	1.80E-08	NEGR1
rs11690172	2	57387094	A	G	0.6119	0.015	0.003	1.99E-08	VRK2
rs2610986	4	18037231	T	C	0.6511	-0.016	0.003	2.01E-08	LCORL
rs895606	9	88003668	A	G	0.4328	0.015	0.003	2.25E-08	AGTPBP1
rs114598875	2	60976384	A	G	0.8246	-0.02	0.004	2.41E-08	PAPOLG
rs1606974	2	51873599	A	G	0.1101	0.022	0.004	2.80E-08	NRXN1
rs2964197	5	57535206	T	C	0.5019	0.015	0.003	3.02E-08	PLK2
rs62379838	5	120102028	T	C	0.681	0.016	0.003	3.30E-08	PRR16
rs34072092	4	28801221	T	C	0.8694	0.024	0.004	3.91E-08	STIM2
rs6739979	2	193731929	T	C	0.597	-0.015	0.003	4.70E-08	PCGEM1
